# Effects of Ammonia Stress on the Antioxidant, Ferroptosis, and Immune Response in the Liver of Golden Pompano *Trachinotus ovatus*

**DOI:** 10.3390/antiox14040419

**Published:** 2025-03-31

**Authors:** Yafei Duan, Meng Xiao, Ruijie Zhu, Yuxiu Nan, Yukai Yang, Xiaohua Huang, Dianchang Zhang

**Affiliations:** 1 State Key Laboratory of Mariculture Biobreeding and Sustainable Goods, Key Laboratory of South China Sea Fishery Resources Exploitation & Utilization, Ministry of Agriculture and Rural Affairs, South China Sea Fisheries Research Institute, Chinese Academy of Fishery Sciences, Guangzhou 510300, China; duanyafei89@163.com (Y.D.);; 2 Key Laboratory of Efficient Utilization and Processing of Marine Fishery Resources of Hainan Province, Sanya Tropical Fisheries Research Institute, Sanya 572018, China

**Keywords:** fish, ammonia, liver, antioxidant, ferroptosis, immunity

## Abstract

Ammonia is the main harmful environmental substance affecting fish culture. The liver is the immune and metabolic organ of fish, and its physiological homeostasis will affect the health of the organism. In this study, healthy golden pompano *Trachinotus ovatus* juveniles were exposed to 5 mg/L (A5) and 10 mg/L (A10) ammonia-N stress for 7 days and then the variation characteristics of the physiological homeostasis of the liver were analyzed at multiple biological levels. After ammonia stress, the liver showed obvious morphological changes and stress responses. Specifically, the oxidative stress indexes, such as the activities of the anti-superoxide anion generation capacity (ASC) and superoxide dismutase (SOD), were elevated in the A5 and A10 groups, while the glutathione peroxidase (GPx) activity and glutathione (GSH) content were disturbed; the relative expression levels of the *Nrf2* and *NQO1* genes were increased in the A10 group, while the expressions of the *Keap1* and *HO1* were decreased in the A5 and A10 groups. Ferroptosis related genes, such as the relative expressions of *NOX1*, *NCOA4*, and *FPN1* were increased in the A5 and A10 groups, *PTGS2* and *FTH1* were decreased in the A5 group but elevated in the A10 group, and *p53*, *GPx4*, *SLC7A11*, and *NFS1* were only increased in the A10 group. Inflammation related genes, such as *TNFα*, *IL1β*, and *IL8* relative expression levels, were increased in the A10 group, *IL10* was increased in the A5 and A10 groups, while *TGFβ* was decreased in the A5 group but increased in the A10 group. Immune related genes, such as the expression levels of *IgM* and *IgT*, were increased in the A5 group but decreased in the A10 group. The integrated biomarker responses revealed that the hepatotoxicity of ammonia was concentration-dependent, and there was a high correlation between oxidative stress, ferroptosis, inflammation, and immune function changes. These results reveal the hepatotoxicity of ammonia stress on *T. ovatus*.

## 1. Introduction

The golden pompano, *Trachinotus ovatus*, is an important marine economic fish; farming output reached a record of 292,263 tons in 2023 in China [1]. *T. ovatus* is mainly cultivated in seawater ponds and floating cages with high density. However, the frequent outbreaks of diseases seriously affect the culture of *T. ovatus*, and environmental stress is one of the main incentives. Ammonia constitutes a predominant toxicant in aquaculture systems, which is mainly produced by unconsumed feed and excreta of aquatic animals [2]. Ammonia is highly toxic and can affect the respiration of fish, lead to metabolic disorder, decreased immunity, and makes it easy to be infected by pathogens [3,4,5,6]. During *T. ovatus* culture, especially in high-density ponds and juvenile fish cultures, rising ammonia will cause many fish deaths and huge economic losses to farmers. However, current research pays little attention to the toxicity of ammonia to *T. ovatus*, and analyzing their physiological response to ammonia stress is conducive to developing corresponding stress prevention strategies.

The toxicity of ammonia stress to *T. ovatus* has been reported in a few studies. For example, the 96-h lethal concentration of ammonia-N of *T. ovatus* juveniles was 26.9 mg/L [5]. After ammonia-N stress for 96 h, plasma biochemical indexes were increased significantly and the antioxidant enzymes activity was also disturbed in the liver of *T. ovatus*; these changes could not return to normal in a short time [5]. Furthermore, ammonia stress induced the transcription level of inflammatory cytokines genes in the gills of *T. ovatus*, and *HIF-1α*/*NF-κB* signaling played a key role in immune response [4]. Ammonia toxicity has also been reported in other fish species. For example, 60.0 mg/L ammonia stress caused liver pathological damage in *Coregonus ussuriensis* and increased the expression of immune-related genes such as tumor necrosis factor-α (*TNFα*), interleukin 1β (*IL1β*), and interleukin 8 (*IL8*) [7]. Ammonia stress can also reduce cell viability and glutathione (GSH) content and increase the reactive oxygen species (ROS) level in the head kidney macrophages of yellow catfish (*Pelteobagrus fulvidraco*) [3]. Ammonia stress induced immunoglobulin M (IgM) and complement 3 levels in the head kidney of silver carp (*Hypophthalmichthys molitrix*) [8].

Ferroptosis is a new type of iron-dependent programmed cell death, which is characterized by iron overload, lipid peroxidation, and antioxidant system imbalance [9]. NADPH oxidase 1 (NOX1) and nuclear receptor coactivator 4 (NCOA4) regulate ferroptosis by aggravating lipid peroxidation [10,11]. Prostaglandin-endoperoxide synthase 2 (PTGS2) is considered as a biomarker of ferroptosis [11]. Solute carrier family 7 member 11 (SLC7A11) is an amino acid carrier that can transport cystine to synthesize GSH, while glutathione peroxidase 4 (GPx4) exerts antioxidant activity by catalyzing GSH decomposition [9]. Cysteine desulfurase (NFS1) can influence ferroptosis by affecting cysteine metabolism [12]. The p53 can inhibit the expression of SLC7A11 to increase the sensitivity of cells to ferroptosis [13]. In iron metabolism, ferritin 1 (FTH1) can store excessive cellular iron, while ferroportin 1 (FPN1) can export intracellular iron to the outside of the cell, which together regulate ferroptosis homeostasis [14]. At present, there are few studies of ferroptosis in fish under ammonia stress. For example, ammonia stress can activate ferroptosis in the liver and brain tissue and head kidney macrophages of yellow catfish *P. fulvidraco* [3,15,16]. In one study, 32.5 mg/L acute ammonia stress induced ferroptosis and mitochondrial damage in the liver of gibel carp (*Carassius gibelio*) [17]. However, there is a lack of research reports on the ferroptosis of *T. ovatus* under ammonia stress.

The liver is an important organ for immunity and metabolism, and its physiological disorder will seriously affect the health of fish [18]. At present, the hepatotoxicity of ammonia stress to *T. ovatus* is still unclear. Therefore, in this study, the golden pompano *T. ovatus* juveniles were separately exposed to 5 and 10 mg/L ammonia-N stress for 7 days, and then the changes of tissue morphology, oxidative stress, ferroptosis, inflammation, and immunity in the liver were explored. In addition, the correlation between different physiological indexes was also analyzed. The results are helpful to clarify the hepatotoxicity of ammonia stress to *T. ovatus*.

## 2. Materials and Methods

### 2.1. Fish Culture Conditions

The *T. ovatus* used in this study were randomly collected from a deep-water cage in Shenzhen (China) and transferred to experimental tanks in the workshop for temporary cultivation. The transshipment process was completed within 20 min. The average weight of the fish was 10.2 ± 1.7 g and the appearance of fish body and gills was normal, with no disease characteristics. The fish were temporarily cultured for 3 d in a culture tank containing 300 L seawater, and the density was 20 fish/300 L. During the temporary period, flowing water was used for each tank to keep the water quality safe for fish, including temperature 29 ± 0.5 °C, pH 7.9–8.0, salinity 30‰, and dissolved oxygen above 6.0 mg/L, which were detected by water quality analysis equipment (YSI, Yellow Springs, OH, USA). The ammonia level in each tank was outside of the detection limit. The fish were fed with commercial compound feed twice a day (8:00 and 17:00), and the uneaten feed and feces were cleaned out in a timely manner.

### 2.2. Ammonia Stress Exposure and Sampling

After the fish were temporarily raised, 180 fish were randomly assigned to 3 treatments: control (CK), 5 mg/L ammonia-N (A5), and 10 mg/L ammonia-N (A10) stress group. There were 3 tanks in each group, and each tank raised 20 fish in 300 L seawater. The CK group was fresh filtered seawater without ammonia-N regulation. The ammonia-N concentration of the rearing water in the A5 and A10 groups were 5 and 10 mg/L, respectively, both of which were regulated by ammonium chloride (Shanghai Macklin Biochemical Technology Co., Ltd., Shanghai, China). The setting of ammonia-N concentration was based on the actual monitoring of fish culture and related research reports [4,5], which were close to 20% and 40% of 96 h LC50 of ammonia-N toxicity to *T. ovatus*, respectively. The concentration of ammonia-N was detected regularly and adjusted in time to maintain the stability of stress concentration. The ammonia-N concentration was measured using the hypobromite oxidation method. The measured ammonia-N concentration of the CK, A5, and A10 groups were 0.03 ± 0.02, 5.13 ± 0.16, and 10.27 ± 0.24 mg/L, respectively. The same tank was used in the temporary culture and stress stage, and all the fish were not transferred. During the exposure period, the seawater of each tank was changed every day, and other culture conditions remained the same as the temporary culture period except the concentration of ammonia-N. The survival and behavior of the fish were observed and recorded in time. The stress exposure lasted for 7 days.

At the 7th day of stress, the liver samples of each tank were randomly collected. In detail, the livers of three fish from each tank were fixed in 4% paraformaldehyde for histological analysis; the livers of three fish from each tank was used for biochemical analysis; and the livers of three fish from each tank was used for gene expression analysis.

### 2.3. Histomorphological Analysis

The liver samples were fixed for 24 h, and then the tissue sections were made. The fixed tissue was washed by running water for 30 min, then were dehydrated in a series of ethanol concentrations (70%, 80%, 90%, and 100%), rinsed with xylene, embedded in paraffin, and cut using a microtome (Leica RM2016, Wetzlar, Germany) into 4 μm slices. After hematoxylin and eosin (H&E) staining, the sections were examined and photographed under a microscope (Nikon, Tokyo, Japan).

### 2.4. Biochemical Analysis

The liver samples were prepared into 10% homogenate with physiological saline, and centrifuged with 1370 g at 4 °C for 15 min. The supernatant was collected and stored for the detection of biochemical index, including total antioxidant capacity (TAC), anti-superoxide anion generation capacity (ASC), total superoxide dismutase (SOD), catalase (CAT), GPx, and GSH. All the biochemical indexes were detected by the commercial kits (Nanjing Jiancheng Bioengineering Institute, Nanjing, China), and analyzed using an enzyme-labeled instrument (Spark, TECAN, Grödig, Austria).

### 2.5. Gene Expression Analysis

The total RNA of the liver was extracted by TRIzol method (Invitrogen, Carlsbad, CA, USA), and the genomic DNA was removed and purified; the cDNA was synthesized using the SweScript RT II First Strand cDNA Synthesis Kit (Servicebio, Wuhan, China). Fluorescence real-time quantitative PCR (qPCR) was performed using the SGExcel Fast SYBR qPCR Mixture Kit (Sangon Biotech, Shanghai, China) on a qPCR system (CG-02, Heal Force, Shanghai, China). The qPCR primer sequences are shown in Table S1, and the *β-actin* gene of *T. ovatus* was used as internal reference. The primers were synthesized and purified by Sangon Biotech (Shanghai, China), and the amplification efficiency of the primers were determined by amplification plots and melting curve. The relative mRNA expression levels were calculated using the method of Livak and Schmittgen (2001) [19]. Detailed methods are presented in Duan et al. (2024) [20].

### 2.6. Integrated Biomarker Response (IBR)

IBR analysis mainly integrates the measured biomarkers into a comprehensive index to evaluate the response of organisms to stress levels [21]. In this study, the biochemical and gene indexes we measured were classified into four categories according to their functions: oxidative stress, ferroptosis, inflammation, and immunity. Then, the comprehensive toxicity of different concentrations of ammonia stress to the liver was analyzed by the IBR index, which was expressed by radar chart. The detailed method is to refer to [7].

### 2.7. Statistical Analysis

The correlation between the changes in all the biochemical indexes and gene expression was analyzed using Pearson coefficient. All the data were expressed as mean ± standard error (SE). The normality and homogeneity of the data were determined using the Shapiro–Wilk test and the Levene test method. Statistical analysis was performed using a one-way analysis of variance (ANOVA) with SPSS 27.0, followed by Tukey post hoc test. *p* < 0.05 was considered as significant difference.

## 3. Results

### 3.1. The Survival of the Fish

At the stage of ammonia stress, none of the fish in all the groups were dead. However, there were some anomalies in the vitality. For example, compared with the CK group, the shoal behavior and swimming speed of the fish of the A5 and A10 groups were weaker.

### 3.2. Histomorphological Changes of the Liver

**Figure 1:**
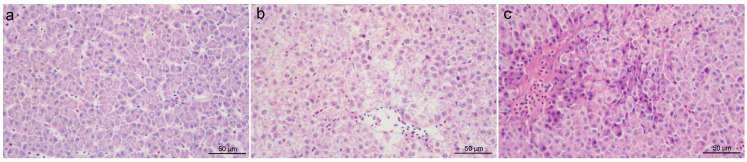
The changes in the liver tissue morphology of *T. ovatus* after ammonia stress. HE (×400): (**a**) control (CK) group; (**b**) 5 mg/L ammonia-N stress (A5) group; (**c**) 10 mg/L ammonia-N stress (A10) group.

The histomorphological changes of the liver were evaluated by HE staining. In the CK group, the liver tissue morphology was relatively normal, and the hepatocytes were arranged neatly and closely (Figure 1a). After ammonia stress, the hepatocytes became hypertrophy, vacuolation, and nucleus deviation or loss (Figure 1b). Especially in the A10 group, the liver cells had unclear contour, serious vacuolation, and even watery degeneration (Figure 1c).

### 3.3. The Oxidative Stress Response in the Liver

**Figure 2:**
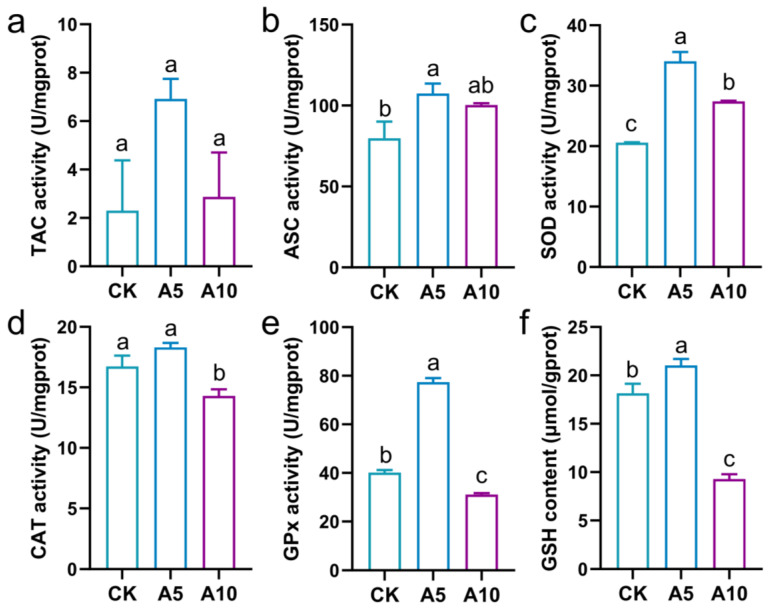
The changes in the biochemical indexes related to oxidative stress in the liver of *T. ovatus* after ammonia stress. (**a**) total antioxidant capacity (TAC) activity; (**b**) anti-superoxide anion generation capacity (ASC) activity; (**c**) total superoxide dismutase (SOD) activity; (**d**) catalase (CAT) activity; (**e**) glutathione peroxidase (GPx) activity; (**f**) glutathione (GSH) content. The lowercase letters on the bar indicate significant differences (*p* < 0.05). CK, control group; A5, 5 mg/L ammonia-N stress group; A10, 10 mg/L ammonia-N stress group.

The redox homeostasis of the liver was explored (Figure 2). Compared with the CK group, the TAC activity was not affected significantly by ammonia stress (*p* > 0.05); the ASC activity was significantly increased in the A5 group (*p* < 0.05); the SOD activity was significantly increased in the A5 and A10 groups (*p* < 0.05); the CAT activity was significantly decreased in the A10 group (*p* < 0.05), but was no significant difference in the A5 group (*p* > 0.05); the GPx activity and the GSH content were significantly increased in the A5 group, but significantly decreased in the A10 group (*p* < 0.05).

**Figure 3:**
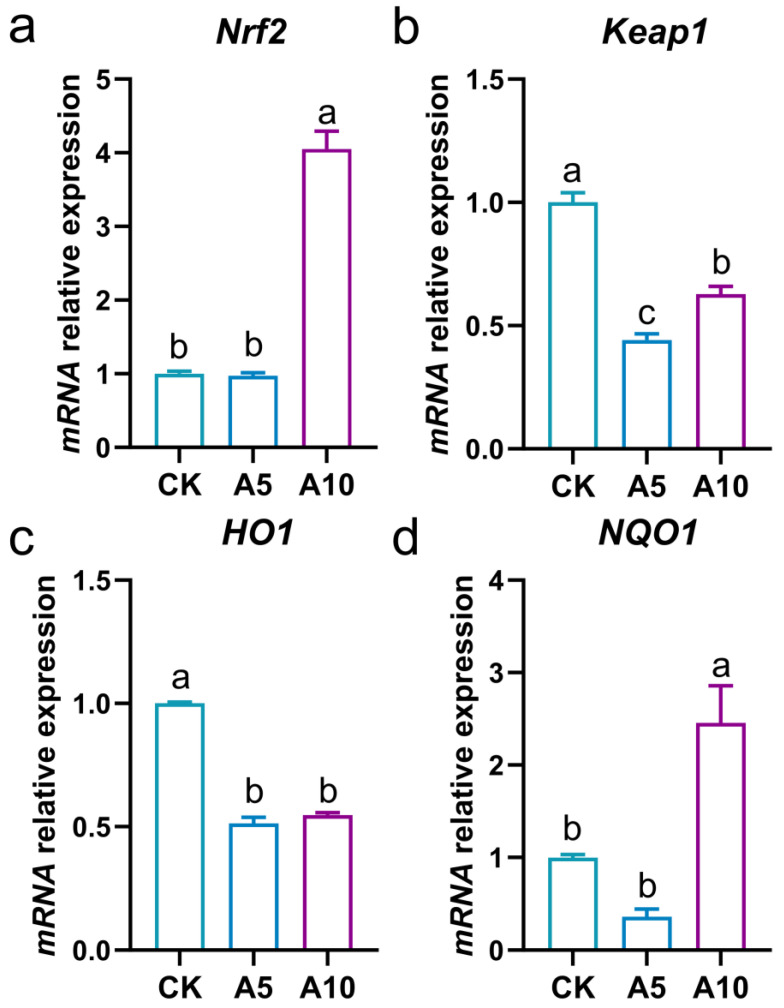
The changes in the mRNA relative expression levels of the antioxidant signaling genes in the liver of *T. ovatus* after ammonia stress. (**a**) *Nrf2* gene expression; (**b**) *Keap1* gene expression; (**c**) *HO1* gene expression; (**d**) *NQO1* gene expression. The lowercase letters on the bar indicate significant differences (*p* < 0.05). CK, control group; A5, 5 mg/L ammonia-N stress group; A10, 10 mg/L ammonia-N stress group.

The antioxidant signaling genes expression in the liver was explored (Figure 3). Compared with the CK group, the relative mRNA expression levels of the nuclear factor erythroid 2-related factor 2 (*Nrf2*) and NAD(P)H quinone oxidoreductase 1 (*NQO1*) genes showed no significant changes in the A5 group (*p* > 0.05), but were significantly increased in the A10 group (*p* < 0.05). The expressions of the kelch-like ECH-associated protein 1 (*Keap1*) and heme oxygenase-1 (*HO1*) genes were significantly decreased in the A5 and A10 groups (*p* < 0.05).

### 3.4. Changes of Ferroptosis-Related Gene Expression in the Liver

**Figure 4:**
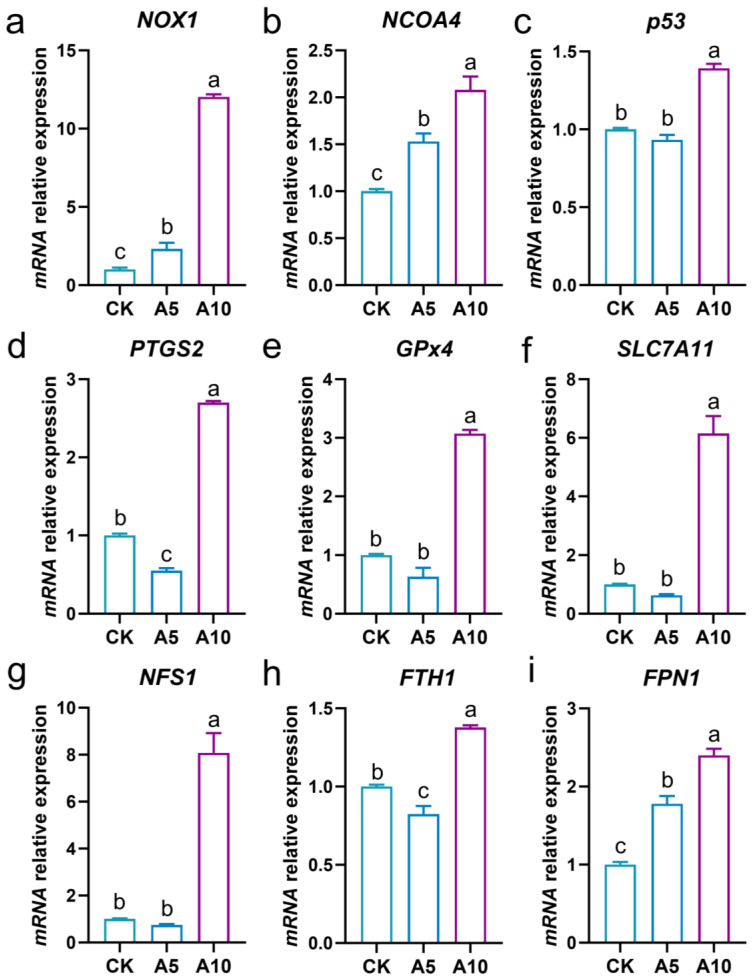
The changes in the mRNA relative expression levels of the ferroptosis related genes in the liver of *T. ovatus* after ammonia stress. (**a**) *NOX1* gene expression; (**b**) *NCOA4* gene expression; (**c**) *p53* gene expression; (**d**) *PTGS2* gene expression; (**e**) *GPx4* gene expression; (**f**) *SLC7A11* gene expression; (**g**) *NFS1* gene expression; (**h**) *FTH1* gene expression; (**i**) *FPN1* gene expression. The lowercase letters on the bar indicate significant differences (*p* < 0.05). CK, control group; A5, 5 mg/L ammonia-N stress group; A10, 10 mg/L ammonia-N stress group.

The ferroptosis homeostasis of the liver was explored (Figure 4). Compared with the CK group, the relative mRNA expression levels of the *NOX1*, *NCOA4*, and *FPN1* genes were significantly increased in the A5 and A10 groups (*p* < 0.05); the expressions of the *p53*, *GPx4*, *SLC7A11*, and *NFS1* genes showed no significant changes in the A5 group (*p* > 0.05), but were significantly increased in the A10 group (*p* < 0.05); the expressions of the *PTGS2* and *FTH1* were significantly decreased in the A5 group, but significantly increased in the A10 group (*p* < 0.05).

### 3.5. Changes of Inflammation-Related Gene Expression in the Liver

**Figure 5:**
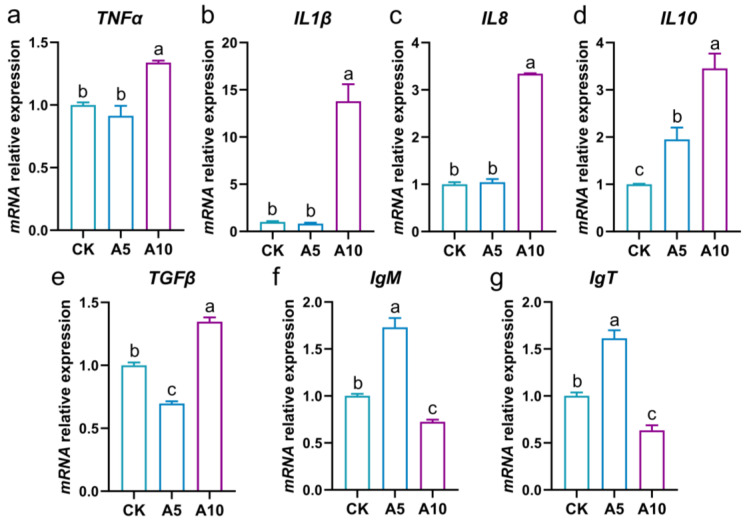
The changes in the mRNA relative expression levels of the inflammatory and immune related genes in the liver of *T. ovatus* after ammonia stress. (**a**) *TNFα* gene expression; (**b**) *IL1β* gene expression; (**c**) *IL8* gene expression; (**d**) *IL10* gene expression; (**e**) *TGFβ* gene expression; (**f**) *IgM* gene expression; (**g**) *IgT* gene expression. The lowercase letters on the bar indicate significant differences (*p* < 0.05). CK, control group; A5, 5 mg/L ammonia-N stress group; A10, 10 mg/L ammonia-N stress group.

The inflammatory response of the liver was evaluated (Figure 5a–e). Compared with the CK group, the relative mRNA expression levels of the *TNFα*, *IL1β*, and *IL8* genes showed no significant changes in the A5 group (*p* > 0.05), but were significantly increased in the A10 group (*p* < 0.05); the expression of interleukin 10 (*IL10*) was significantly increased in the A5 and A10 groups (*p* < 0.05); the expression of the transforming growth factor-*β* (*TGFβ*) was significantly decreased in the A5 group, but significantly increased in the A10 group (*p* < 0.05).

### 3.6. Changes of Immune-Related Gene Expression in the Liver

The immune response of the liver was evaluated (Figure 5f,g). Compared with the CK group, the relative mRNA expression levels of the *IgM* and immunoglobulin T (*IgT*) were significantly increased in the A5 group, but significantly decreased in the A10 group (*p* < 0.05).

### 3.7. Integration of the Multi-Level Biomarker Responses

**Figure 6:**
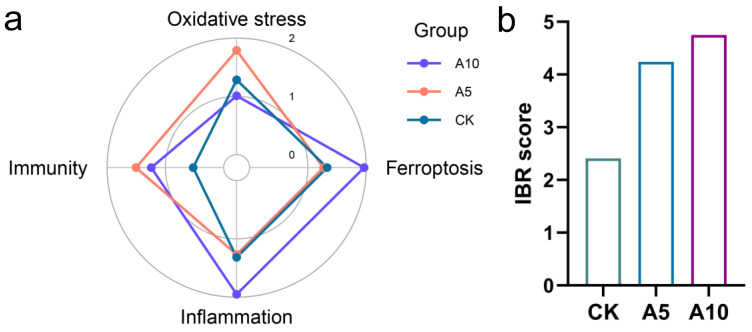
The IBR index analysis of the biomarker responses in the liver of *T. ovatus* after ammonia stress. (**a**) The star plots of biomarker responses; (**b**) The IBR scores. CK, control group; A5, 5 mg/L ammonia-N stress group; A10, 10 mg/L ammonia-N stress group.

The effects of ammonia stress on different physiological functions of the liver are shown in the IBR star plots (Figure 6a). Among them, the oxidative stress index was more affected in the A5 group, while ferroptosis and inflammation were more affected in the A10 group. Additionally, immune indexes were affected to varying degrees in the A5 and A10 groups. The IBR score showed that with the increase of ammonia concentration, the degree of hepatotoxicity was increased (Figure 6b).

### 3.8. The Correlation Between the Changes of the Biomarkers

**Figure 7:**
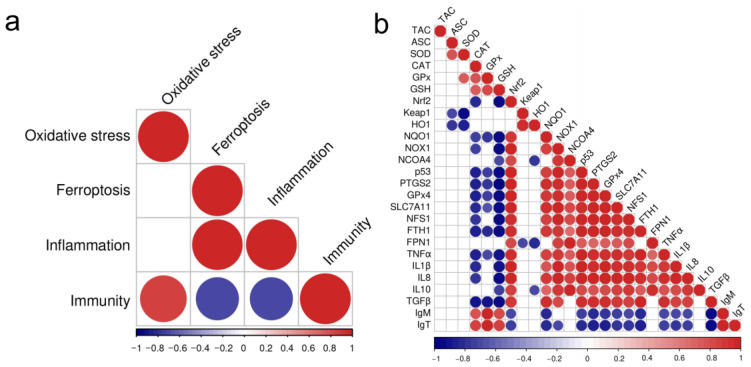
The correlation analysis of the changes of physiological indexes in the liver of *T. ovatus* after ammonia stress. (**a**) The correlation between the different physiological functions; (**b**) The correlation between the different physiological indexes. The color of the circle represents correlation: the red is positive correlation and the blue is negative correlation.

Furthermore, a correlation analysis was performed on different physiological function changes (Figure 7a). Among them, the change of oxidative stress index was positively correlated with immunity; the changes of ferroptosis index were positively correlated with inflammation, but negatively correlated with immunity; and the change of inflammatory index was negatively correlated with immunity.

The correlation between different physiological indexes was further revealed (Figure 7b). The oxidative stress indexes (i.e., CAT, GPx and GSH) were negatively correlated with the changes of ferroptosis (i.e., *p53*, *PTGS2*, *GPx4*, *SLC7A11*, and *FTH1*), but were positively correlated with the changes of immunity (*IgM* and *IgT*). The ferroptosis indexes were positively correlated with the changes of inflammation (i.e., *TNFα*, *IL1β*, *IL8*, and *IL10*). The proinflammatory cytokines (i.e., *TNFα*, *IL1β*, and *IL8*) were negatively correlated with the changes of immunity (*IgM* and *IgT*).

## 4. Discussion

Ammonia is a key environmental factor endangering fish culture. However, the mechanism of hepatotoxicity of ammonia stress to golden pompano *T. ovatus* is still unclear. In this study, after 7 days of ammonia stress, no death of *T. ovatus* was observed in all the groups, but their shoal behavior and swimming speed were weaker. Histological sections based on the HE staining showed obvious changes in the liver morphology, which meant that its physiological homeostasis was at risk of being disturbed. Therefore, we explored the physiological response characteristics in the liver of *T. ovatus* to ammonia stress at different biological levels.

Oxidative stress is one of the main toxicities of environmental pollutants to fish. The antioxidant system of the organism can eliminate the excessive ROS induced by stress to resist oxidative damage. Of these, TAC can reflect the overall antioxidant capacity of the organism, while antioxidant enzymes such as SOD, CAT, and GPx can jointly decompose ROS [22,23]. GSH is a compound with antioxidant and detoxification properties [24]. In the present study, after ammonia stress, the TAC, ASC, and SOD activities were increased, and the CAT and GPx activities and the GSH content were disordered, which revealed that ammonia stress caused oxidative stress and interfered with redox homeostasis in the liver. Nrf2 is a core transcription factor in the antioxidant pathway, with Keap1 as its negative regulator [25]; HO1 and NQO1 are downstream effector proteins after Nrf2 activation, playing crucial roles in the cellular antioxidant defense system [26]. In the present study, after ammonia stress, the *Nrf2* and *NQO1* genes expression were induced in the A10 group, while the *Keap1* and *HO1* genes expression were deduced in the A5 and A10 groups. This phenomenon once again confirmed that ammonia stress affected the antioxidant homeostasis in the liver of *T. ovatus*.

Oxidative stress is an important inducer of programmed cell death (PCD). Ferroptosis is a new iron-dependent PCD pattern, which is precisely regulated by cellular metabolic networks such as lipid, iron, and amino acid metabolism and participates in the pathogenesis of diseases [11]. In the occurrence of ferroptosis, NCOA4, p53, NOX1, and PTGS2 are the main inducers, while GPx4, SLC7A11, NFS1, FTH1, and FPN1 are inhibitors [11,27,28]. In the present study, the increased expression levels of the *NOX1*, *NCOA4*, *p53*, and *PTGS2* genes indicated that ammonia stress triggered the ferroptosis process in the liver of *T. ovatus*. Futhermore, the increases of the *GPx4*, *SLC7A11*, and *NFS1* genes expression in the A10 group were conducive to the defense against the damage caused by ferroptosis. FTH1 and FPN1 are two functional proteins that regulate the homeostasis of iron metabolism in cells [14]. In the present study, the disordered expression of the *FTH1* and *FPN1* genes indicated that ammonia stress caused ferroptosis in the liver of *T. ovatus* by interfering with iron metabolism homeostasis. Therefore, we concluded that ammonia stress induced ferroptosis in the liver of *T. ovatus* with an obvious toxic concentration effect.

The aquatic animals are often accompanied by inflammation when they suffer from environmental stress. Among them, the pro-inflammatory cytokines such as IL1β, IL8, and TNFα can activate inflammatory responses, while IL10 mainly exerts anti-inflammatory effect [29]. TGFβ participates in the regulation of inflammatory process [30]. In the present study, the expression levels of the *TNFα*, *IL1β*, and *IL8* genes were increased in the A10 group, indicating that inflammatory response was induced in the liver of *T. ovatus*. In addition, the expression of the *IL10* gene was increased in both stress groups, while that of the *TGFβ* gene was deduced in the A5 group but induced in the A10 group. This phenomenon revealed that anti-inflammatory molecules in the liver were induced, but not enough to defend against the inflammatory response.

Immunoglobulin is an important immune molecule in fish that contributes to the defense against infection and stress [31,32]. There are three main types of Ig in teleost, including IgM, IgD, and IgT. Among them, IgM is the main immunoglobulin of fish and plays a key role in systemic adaptive immunity [33]. The levels of the IgM were significantly increased in the head kidney of *H. molitrix* after 24 and 48 h of ammonia stress [8]. In the present study, after ammonia stress, the expression levels of the *IgM* gene were increased in the A5 group but decreased in the A10 group. IgT is a unique immunoglobulin of teleost, which is mainly expressed in lymphoid tissues [33]. In grouper, the *IgT* gene was expressed in the head kidney, followed by the spleen, and low expression was seen in the gills, thymus, gut, and liver [34,35]. In the present study, after ammonia stress, the expression change of the *IgT* gene was consistent with that of the *IgM* gene. This phenomenon revealed that when the fish were exposed to 5 mg/L ammonia-N stress, the liver immune response was activated to defend against stress, while the liver immune function was decreased in the fish exposed to 10 mg/L ammonia-N stress.

As an important toxic effect evaluation method, the IBR index can reflect the overall biotoxicity response of organisms to environmental stress. In previous studies, Xu et al. (2024) used IBR index to study the toxic effects of acute ammonia stress on ussuri cisco (*C. ussuriensis*) [7]. In the present study, based on the IBR index analysis, the toxicity of ammonia stress to the liver of *T. ovatus* increased with the elevation of its concentration. Furthermore, we found that the oxidative stress indexes were positively correlated with the immune indexes, indicating that the oxidative stress induced by ammonia stress might further affect the immunity of the liver. In addition, the changes of the ferroptosis function were positively correlated with the inflammation, but negatively correlated with the immunity, indicating that the ferroptosis induced by ammonia stress might further activate the inflammatory response and disturb immune function in the liver. In the correlation of physiological indexes, we further found that antioxidant indexes (CAT, GPx, GSH) were negatively correlated with ferroptosis indexes (*p53*, *PTGS2*, *GPx4*, *SLC7A11*, *FTH1*). Therefore, we inferred that oxidative stress induced by ammonia stress was related to the ferroptosis homeostasis. Long et al. (2023) reported that in the initial stage of ammonia stress, ferroptosis and inflammation were first activated in the brain of yellow catfish (*P. fulvidraco*) and then oxidative stress was induced [15]. However, in the current research, the relationship between the ferroptosis, oxidative stress, and inflammation in the liver of *T. ovatus* after ammonia stress is still unknown, and needs to be explored in the future.

## 5. Conclusions

**Figure 8:**
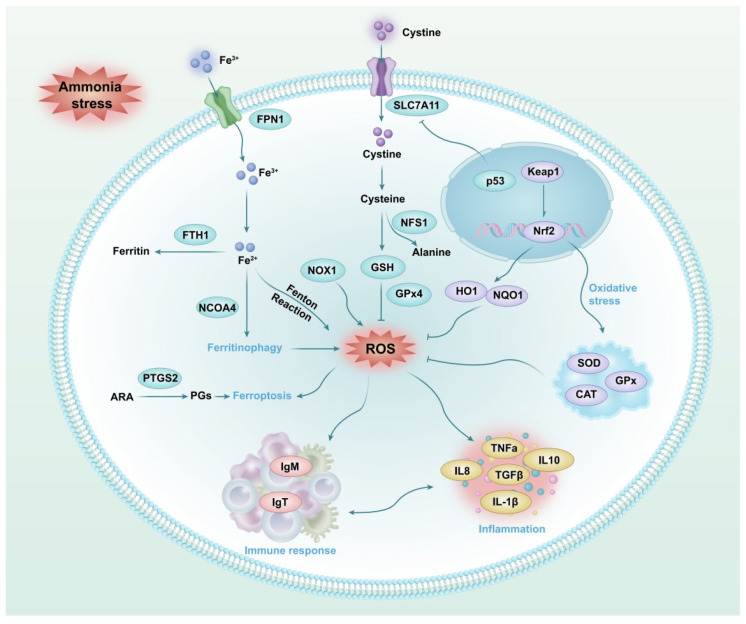
The deduced mechanism of the hepatotoxicity of ammonia stress on the physiological homeostasis of *T. ovatus.* ARA, Arachidonic acid; PGs, Prostaglandin.

This study revealed the toxic effect of ammonia stress on the physiological homeostasis of the liver of *T. ovatus*. Specifically, ammonia stress caused histomorphological changes in the liver, induced oxidative stress, and interfered with antioxidant function through *Nfr2*/*HO1*/*NQO1* signaling and antioxidant enzymes. Furthermore, ammonia stress also influenced ferroptosis homeostasis in the liver by affecting GSH synthesis, iron transport, and ferritin synthesis. Ferritinophagy related gene expressions induced inflammatory response by affecting the expression of interleukin, *TNFα*, and *TGFβ* genes and caused immune dysfunction by interfering with *IgM* and *IgT* gene expression (Figure 8). These findings provide critical insights into the physiological mechanisms of ammonia-induced hepatotoxicity in *T. ovatus* and also facilitate the development of anti-stress strategies.

## Data Availability

Data will be made available upon request.

## Supplementary Materials

The following supporting information can be downloaded at: https://www.mdpi.com/article/10.3390/antiox14040419/s1, Table S1: The primer sequences used in this study.
