# Supplementary material for: Effects of Ammonia Stress on the Antioxidant, Ferroptosis, and Immune Response in the Liver of Golden Pompano Trachinotus ovatus
Source: Antioxidants (Basel). 2025 Mar 31;14(4):419. doi: 10.3390/antiox14040419 (PMC12024250; doi:10.3390/antiox14040419)
Supplement: Supplementary file 1 [file antioxidants-14-00419-s001.zip › antioxidants-3529021-supplementary.pdf]

Table S1 The primer sequences used in this study.

Primers name	Sequences (5′-3′)	prodSize (bp)	Reference
Nrf2-F	AGCTTGGCCTTCATCAAAT	Unpublished	Fish and Shellfish Immunology 130 (2022) 31-42
Nrf2-R	GAGTATGGCTGTCCTTCTTCA		
Keap1-F	AGAGGATGGAGATGGCACAG	Unpublished	Fish and Shellfish Immunology 130 (2022) 31-42
Keap1-R	CATTGGTTGGTCTTGGGATT		
HO1-F	AGAAGATTCAGACAGCAGCAGAACAG	Unpublished	Chemosphere 253 (2020) 126654
HO1-R	TCATACAGCGAGCACAGGAGGAG		
NQO1-F	TGGTCCAGGTGTCACGTCTTCC	Unpublished	Chemosphere 253 (2020) 126654
NQO1-R	GACTTGGCGGTGTAGTGCTTGG		
NOX1-F	GCGTGCCAGCTCAACTCAGG	85	*Trachinotus ovatus* genome
NOX1-R	AGGGACAGACAGAGCCACAGTG		
NCOA4-F	AAGATCGACGAATCAGAGTTCACCATC	108	*Trachinotus ovatus* genome
NCOA4-R	GTCTGACACAGTCTCCATCGCATC		
p53-F	ACAACCACTCAGATCAATCGCTTCC	99	*Trachinotus ovatus* genome
p53-R	GGAGCCAAGACAATGACAGCCTAC		
PTGS2-F	TGTCCCGTGGCGTCCTTCC	80	*Trachinotus ovatus* genome
PTGS2-R	CCTGAGTGAGACGTGCTTGAGTTG		
GPx4-F	GACAGATATTGATGGCAACGTGGTTTC	111	*Trachinotus ovatus* genome
GPx4-R	GCTGCTGGTGGACTTGTGATGG		
SLC7A11-F	GCTAGACCACTACAGTCCATCACAAC	110	*Trachinotus ovatus* genome
SLC7A11-R	TGCTGCAACACTTCCATCACTCTG		
NFS1-F	ACCTCCCAGTCCAGCAGAACG	81	*Trachinotus ovatus* genome
NFS1-R	TGTGTGACCTGTGTGTAACTGTGTG		
FTH1-F	CCGCCGCCGCAAGTGTG	102	*Trachinotus ovatus* genome

FTH1-R	TGACAGACCCAGGACTATTGTGAGG		
FPN1-F	CGTCCAGAACAGTTGCGTCATC	103	GenBank OM643386
FPN1-R	AGTTGTCAGAATCCATCCATTGTAGAG		
TNFα-F	CGCAATCGTAAAGAGTCCCA	Unpublished	Fish and Shellfish Immunology 130 (2022) 31-42
TNFα-R	AAGTCACAGTCGGCGAAATG		
IL1β-F	GCGATTTGGTGCGATTTCT	Unpublished	Fish and Shellfish Immunology 130 (2022) 31-42
IL1β-R	CTCTACTGGCTTGTTGTCTTGC		
IL8-F	GAGAAGCCTGGGAATGGA	136	GenBank KT922006
IL8-R	GAGCCTCAGGGTCTAAGCA		
IL10-F	CGTCCTGGCTCTCTTGTCTCTCCTC	111	GenBank KY231908
IL10-R	TGTCCATGTCATTGTTTGCCTCATA		
TGFβ-F	TATCCCTCTACAACAGCACCA	Unpublished	Fish and Shellfish Immunology 130 (2022) 31-42
TGFβ-R	GGTCAGCAGGCGGTAATC		
IgM-F	TGCTGGCACCCCTTTTACAT	Unpublished	Aquaculture 576 (2023) 739835
IgM-R	CCTCCCAAAAAATTCTCTCAACAG		
IgT-F	TGCCAGTGGGACCAGCCT	Unpublished	Aquaculture 576 (2023) 739835
IgT-R	TGGAAGATCCGCCATGAGTTACT		
β-actin-F	TGAACCCCAAAGCCAACAGG	105	GenBank KX987228
β-Actin-R	CCGCAGGACTCCATACCAAG		
